# The Lattice Distortion, Defect Evolution and Electrochemical Performance Improvement in Zn-VO_2_(B) Nanorods

**DOI:** 10.3390/nano12183196

**Published:** 2022-09-14

**Authors:** Dewei Liu, Qijie Zhang, Xiaohong Chen, Penggang Zhu, Fufeng Yan, Xuzhe Wang, Haiyang Dai, Jing Chen, Gaoshang Gong, Cui Shang, Luogang Xie, Xuezhen Zhai

**Affiliations:** Henan Key Laboratory of Magnetoelectronic Information Functional Materials, School of Physics and Electronic Engineering, Zhengzhou University of Light Industry, 136 Kexue Road, Zhengzhou 450002, China

**Keywords:** cathode materials, positron annihilation spectroscopy, microdefects, lattice distortion, electrochemical performances

## Abstract

Cathode materials of energy storage batteries have attracted extensive attention because of the importance in deciding the rate performance and long cycle property of batteries. Herein, we report a simple and environmentally friendly solvothermal method to prepare Zn-doped VO_2_(B) cathode materials. The introduction of zinc ions can effectively regulate the lattice structure, surface morphology and internal defect state of Zn-VO_2_(B) nano materials. The sample with Zn content *x* = 1.5% has smaller cell volume and grain size, and higher concentration of vacancy defects. These microstructures ensure the structural stability during ion embedding process and, thus, this sample shows excellent electrochemical performances. The capacitance retention rate still maintains 88% after 1000 cycles at the current density of 0.1 A·g^−1^. The enhanced performances of Zn-doped VO_2_(B) samples may lay a foundation for the improvement of electrochemical performances of VO_2_(B) cathode materials for energy storage batteries in the future.

## 1. Introduction

The development of green, sustainable and environmentally friendly energy storage technologies was considered to be an effective way to alleviate the energy crisis and environmental pollution [1]. As the most promising electrochemical energy storage devices, energy storage batteries (lithium ion battery, sodium ion battery, potassium ion battery, zinc ion battery, etc.) and supercapacitors have attracted widespread attention for academia studies and industrial applications [2,3,4]. Throughout the development history of all kinds of energy storage batteries, cathode materials, as one of the key factors affecting the rate performance and long cycle property of batteries, determined the capability of electrochemical energy storage devices [5,6]. Therefore, the selection and structural optimization of cathode materials have become the main research area recently.

Vanadium oxides have gained extensive attention due to their widely variable oxidation states (II-V) providing much wider potential windows and much higher theoretical specific capacity [7]. Among various vanadium oxides, monoclinic phase VO_2_(B) stands out because of the unique layered structure formed by corners and/or edge-sharing VO_6_ octahedra, which supports the intercalation and deintercalation of Mg^2+^ (0.66 Å), Li^+^ (0.69 Å), Zn^2+^ (0.74 Å), Na^+^ (1.02 Å) and K^+^ ions (1.38 Å) [8,9]. Therefore, VO_2_(B) has been considered as one of the most promising cathode electrode materials of storage batteries [10]. However, VO_2_(B) cathode materials suffer from the reduction of specific capacity and poor cyclic stability due to the disproportionation reaction of V^4+^ ions in the charge and discharge process [11]. Thus, the design of superior VO_2_(B) cathode materials for high-performance energy storage battery is highly desirable but much more challenging.

Nanostructured materials have unique advantages in energy storage, such as shorter ion diffusion distance, larger surface area and more reactive active sites [12,13]. Ion doping can not only efficiently regulate the microstructure of electrode material but also facilitate ionic/electronic conductivity and provide more active sites. Therefore, researchers are committed to synthesizing electrode materials with different nanostructures by optimizing the preparation process. For example, Li et al. [14] prepared W-doped VO_2_(B) nanosheets as the cathode material for water-based zinc ion batteries. The introduction of W ion expanded the lattice distance of VO_2_(B) and accelerated the diffusion rate of zinc ion in the electrochemical reaction process, and thus enhanced the structural stability of the material. The specific capacity retention rate of W-doped VO_2_(B) cathode material was 76.4% after 1000 cycles at the current density of 4 A·g^−1^. Liu et al. [15] introduced Mn ions and polyvinylpyrrolidone (PVP) into the VO_2_(B) system and prepared MnVO_2_-PVP composite with the morphology of nanoprisms and nanosheets. The material had faster ionic diffusion kinetics and better long-term cyclic stability. The specific capacity of synthesized MnVO_2_-PVP was 176.5 mA·h·g^−1^ after 5000 cycles at the current density of 10 A·g^−1^. It also had high energy density (179 w·h·kg^−1^) and power density (7000 W·kg^−1^). N-doped nano-VO_2_(B) cathode material prepared by Lv et al. [16] had many active sites and high conductivity and showed excellent electrochemical performances. Compared with carbon coating and organometallic hybridization [6,17], which were used to improve the surface area and specific capacitance of VO_2_, ion doping can not only regulate the crystal structure and electron distribution state in materials, but also introduce moderate amount of defects by controlling the doping concentration, which is convenient to study the evolution of defects.

As an important part of microstructures, microdefects are a deep factor affecting the electrochemical performances of cathode materials. By adjusting the internal microdefects of materials, the local density of electronic states of metal ions can be effectively regulated, the intrinsic conductivity of materials can be improved and more active sites can be provided for the embedding of carriers, thus promoting the embedding and de-embedding of metal ions. In recent years, researchers have also made some explorations on the microdefects of VO_2_(B) cathode materials. Li et al. [18] confirmed through density functional theory analysis that oxygen vacancies could reduce the band gap of VO_2_(B) and the energy barrier of zinc ions diffusion, which were conducive to improving the reaction kinetics and the storage capacity of zinc ions in VO_2_(B) cathode materials. Zhang et al. [19,20] developed VO_2_(B) rich in oxygen vacancies as the cathode material of water-based zinc ion battery. Due to the existence of oxygen defects, the diffusion of zinc ion in an aqueous Zn/VO_2−*x*_(B) battery was significantly enhanced and then the electrochemical performance was improved. The above results show that the defects have important effects on the electrochemical properties of cathode materials, which play a good guiding role in the experimental study of this project. Through simulation calculation, our research group found that there are many kinds of vacancy defects in the VO_2_(B) crystal structure, and positron annihilation lifetime spectrum can be used to determine the type of vacancy defects. In our previous studies, positron annihilation and other techniques were used to study the microdefects and electrochemical properties of nano-VO_2_(B) cathode materials doped with magnesium, manganese and other ions, and it was found that vanadium vacancy is one of the important factors affecting the electrochemical properties of the materials [21,22].

Inspired by the previous works, we successfully constructed Zn-doped VO_2_(B) nanorods as cathode material of storage batteries. The crystal structure, surface morphology, microdefects and electrochemical performances of Zn-VO_2_(B) samples with different Zn doping concentration *x* had been studied comprehensively. Under the combined actions of lattice distortion and microdefects, the Zn-VO_2_(B) sample with doping concentration *x* = 1.5% had a larger specific capacitance and significantly improved electrochemical performances. These results demonstrated that Zn doping was an efficient way to improve the electrochemical performances of VO_2_(B) nanorods, which could promote the application of VO_2_(B) cathode materials in storage batteries.

## 2. Materials and Methods

**Figure 1:**
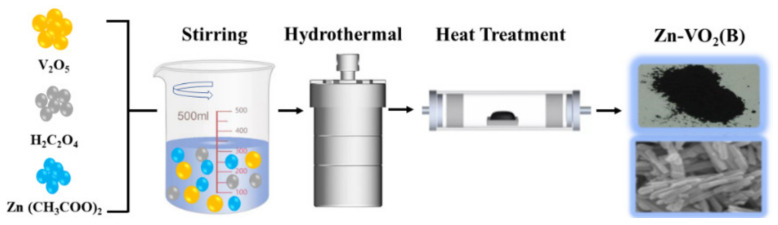
The schematic diagram of the preparation process of the Zn-VO_2_(B).

The Zn-VO_2_(B) nanorods with the doping content *x* = 0.0%, 0.5%, 1.5% and 3.0% were prepared by a solvothermal reaction method using V_2_O_5_ (99.99%), H_2_C_2_O_4_ (99.0%) and Zn(CH_3_COO)_2_·2H_2_O (99.99%) as starting raw materials. For each composition, the starting materials were weighed in required molar ratios and dissolved in the corresponding solvent. The V_2_O_5_ was dissolved in H_2_C_2_O_4_ (99.0%) according to mole ratio 1:3. The Zn(CH_3_COO)_2_·2H_2_O (99.99%) was dissolved in the mixture of H_2_O_2_ (30.0%) and deionized water. The two mixed solutions were put into the reaction kettle and heated up to 180 °C for 48 h. The sediment was washed with deionized water and anhydrous ethanol and then dried in a vacuum for 10 h at 80 °C. The sediment was annealed at a tubular furnace in a vacuum environment by a two-stage heating program: from room temperature to 200 °C for 2 h and then increased to 350 °C for 6 h with a heating rate of 3 °C/min. The schematic diagram of the preparation process was shown in Figure 1.

The crystal structure and morphology of the prepared samples were characterized by X-ray diffraction (XRD, Bruker D8 Advance, Cu Kα radiation: *λ* = 1.5418 Å) and field-emission scanning electron microscope (FESEM, Quanta 250 FEG), respectively. The elemental composition and valence states of the prepared samples were measured by X-ray photoelectron spectroscopy (XPS, Escalab 250XI). The positron annihilation lifetime spectra of the samples were measured using a fast-fast coincidence lifetime spectrometer with 13 *μ*Ci ^22^Na positron source at room temperature. Furthermore, the lifetime spectra were analyzed by the PATFIT program.

The electrochemical properties of the samples were performed on a CHI760E electrochemical workstation. The working electrode was obtained by mixing 80 wt% active material (Zn-VO_2_), 10 wt% acetylene black and 10 wt% PTFE on nickel foam. Pt metal electrode and saturated calomel electrode were used as counter electrode and reference electrode, respectively. The electrolyte was 2 mol/L KOH solution.

## 3. Results and Discussion

### 3.1. XRD

**Figure 2:**
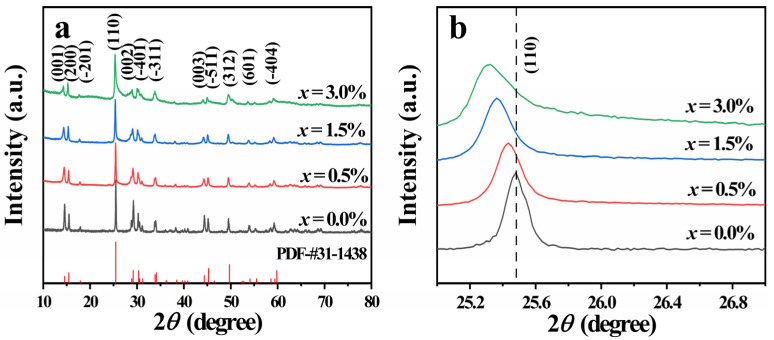
(**a**) XRD patterns of Zn-VO_2_(B) samples with different *x* values; (**b**) magnified patterns around 2*θ* = 25°.

Figure 2a showed the XRD patterns of Zn-VO_2_(B) samples for *x* = 0.0%, 0.5%, 1.5% and 3.0%. The properties (e.g., the phase composition, purity and crystallinity) of the synthesized samples were clearly presented in Figure 2a. The Bragg reflections of all samples were indexed to the monoclinic structure of VO_2_(B) (PDF-# 31-1438) in *C*2/*m* space groups, and no traces of any obvious impurity phases and secondary phases were detected within the detection limits of the instrument. As shown in Figure 2b, with the increasing of Zn content, the (110) peak started to widen and weaken, and shifted towards to small angle side, which was due to the larger ionic radius of doped Zn^2+^ (0.88 Å) compared to that of V^4+^ (0.58 Å). The introduction of Zn ions resulted in an expansive lattice and distorted crystal structure in the samples.

Table 1 illustrated the variation of lattice parameters (a, b, c) and unit cell volumes (V) with doping concentration *x*. Compared with the undoped sample, the lattice constants and cell volume of all doped samples were increased, which was attributed to the lattice expansion caused by large radius ion doping. It was worth noting that the lattice parameters and cell volume did not increase linearly with the increase of doping amount, but showed a nonlinear change with the *x*, namely, decreased with *x* between 0.5% and 1.5%, and then had a sharp increase over 1.5%. The irregular variation was attributable to the distortion of VO_6_ octahedral caused by Zn^2+^ doping [23].

### 3.2. XPS

**Figure 3:**
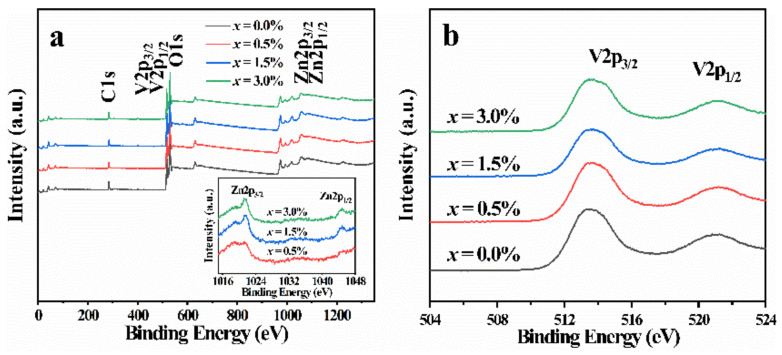
(**a**) X-ray photoelectron full-scan spectrum of Zn-VO_2_(B) samples, illustrations is X-ray photoelectron spectra of Zn 2p_3/2_ and Zn 2p_1/2_; (**b**) X-ray photoelectron spectra of V 2p_3/2_ and V 2p_1/2_.

The chemical microenvironment of the Zn-VO_2_(B) samples was identified by X-ray photoelectron spectroscopy (XPS). As shown in Figure 3a, the XPS full survey spectra presented C, V, O, and Zn element signals. The presence of the elements C was due to the sample contamination. The high-resolution spectrum of Zn 2p showed two peaks at 1021.38 and 1044.78 eV, corresponding to Zn 2p_3/2_ and Zn 2p_1/2_, respectively, as depicted in the illustration of Figure 3a. As shown in Figure 3b, the peaks at 513.7 and 520.9 eV were derived from V^4+^ in VO_2_(B). XPS analysis showed that the Zn atom had been doped into VO_2_(B) successfully and the valence state of V atom did not change during the preparation.

### 3.3. SEM

**Figure 4:**
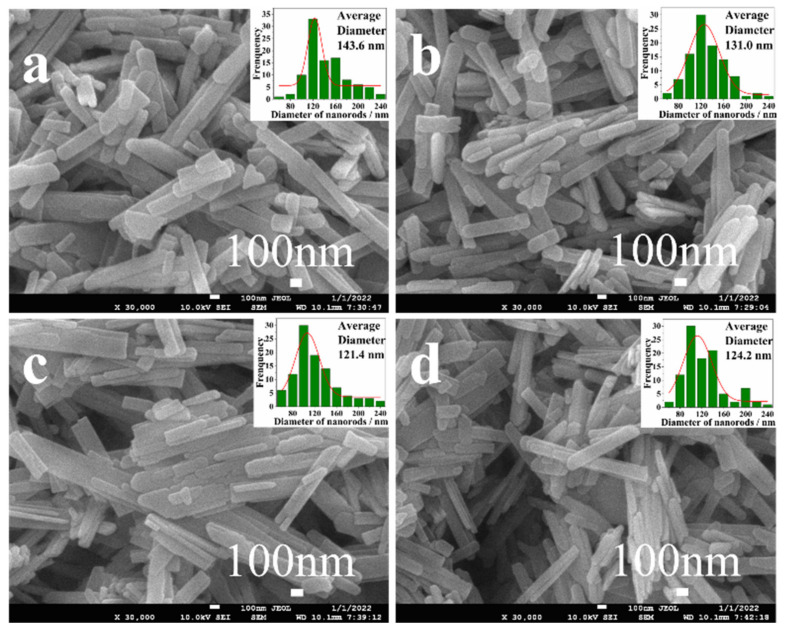
The SEM images of Zn-VO_2_(B) samples: (**a**) *x* = 0.0%; (**b**) *x* = 0.5%; (**c**) *x* = 1.5%; (**d**) *x* = 3.0%.

As shown in Figure 4, the microscopic morphologies of all Zn-VO_2_(B) samples were nanorods. The average width of Zn-VO_2_(B) nanorods was in the range of 120–145 nm. It was noteworthy that the average width of Zn-doped VO_2_(B) nanorods had some changes with the increasing of Zn contents. The average width of Zn-VO_2_(B) nanorods firstly decreased (*x* < 1.5%) and then increased (*x* > 1.5%), and the smallest average width size was 121.4 nm at *x* = 1.5%. The above analysis indicated that Zn doping could regulate the average width of Zn-VO_2_(B) nanorods. Moreover, an appropriate amount of Zn doping may introduce defects in the VO_2_(B) lattice, resulting in the inhibition of grain growth of VO_2_(B) crystals [24].

### 3.4. Positron Annihilation Analysis

Positron annihilation lifetime spectroscopy was performed to characterize the defects in the Zn-VO_2_(B) samples. The positron annihilation lifetime spectra were decomposed into three lifetime components (*τ*_1_, *τ*_2_, *τ*_3_) with corresponding intensities (*I*_1_, *I*_2_, *I*_3_). The longest lifetime *τ*_3_ with lowest intensity *I*_3_ (less than 1.4%) was over 1140 ps, which was ascribed to the annihilation of positrons at the surface of the Zn-VO_2_(B) samples. The discussion of *τ*_3_ and *I*_3_ could be ignored due to the extremely low proportion, and *I*_1_ and *I*_2_ were normalized again. The short lifetime *τ_1_* distributed between 220 and 256 ps was related to the annihilation of the positrons in the perfect lattice and shallow capture region of the experimental samples. The long lifetime *τ*_2_ distributed between 355–434 ps was associated with the positron annihilation in the defect area and represented the size of the vacancy-type defects in the system. The intensity *I*_2_ reflected the concentration of vacancy defects in the experimental samples. For positron experiment, the positron bulk lifetime (*τ*_b_) reflected the nature of the crystal lattice. According to the trapping model, the bulk lifetime *τ*_b_ was given by the following formula [25].

$$\tau_{b}=\frac{1}{\frac{I_{1}}{\tau_{1}}+\frac{I_{2}}{\tau_{2}}}$$

**Figure 5:**
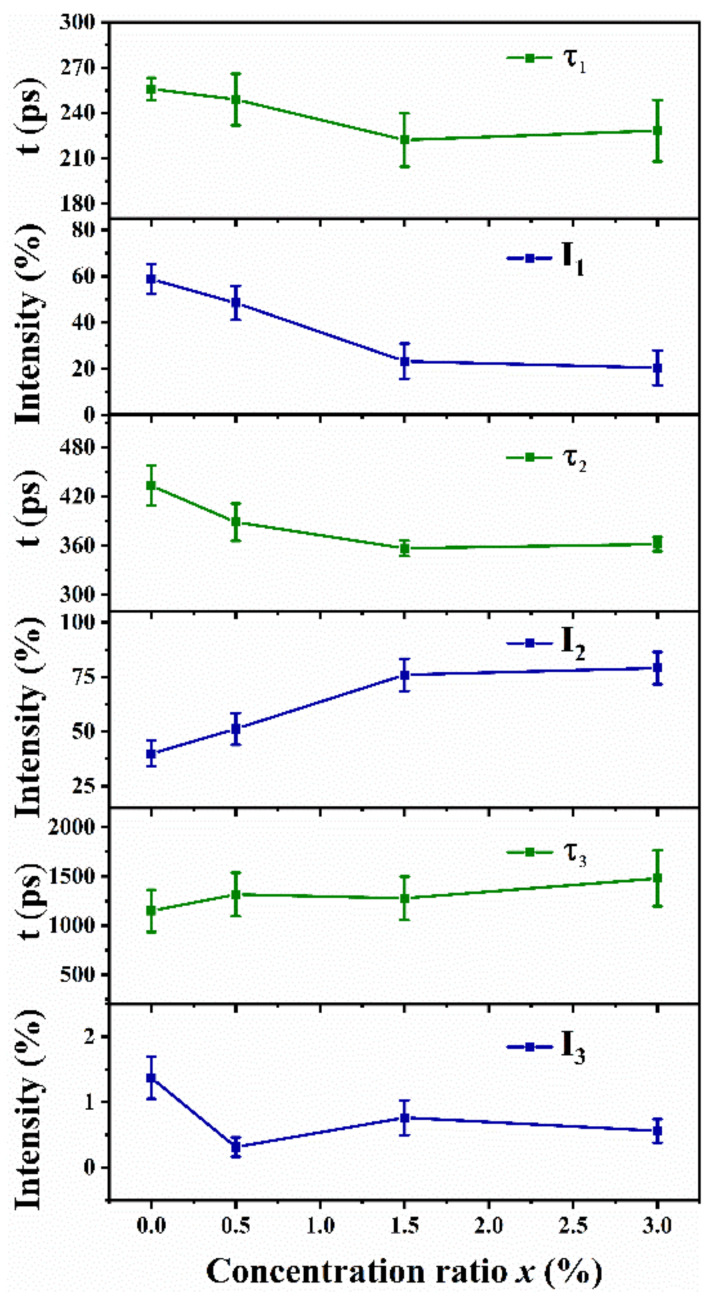
The positron lifetime spectra *τ*_1_, *τ*_2_, *τ*_3_ and *I*_1_, *I*_2_, *I*_3_ of Zn-VO_2_(B) with different *x* values.

The positron lifetime parameters *τ*_1_, *τ*_2_, *τ*_3_, *τ*_b_ and *I*_1_, *I*_2_, *I*_3_ of Zn-VO_2_(B) samples were displayed in Figure 5 and Table 2.

The values of *τ*_b_ had a slight change, which was mainly attributed to the structural distortion caused by Zn doping and the inhomogeneous stress distribution inside the lattice. The value of *τ*_2_ decreased significantly with an increasing Zn doping amount until *x* = 1.5%, whereas it had a moderate increase as *x* > 1.5%. The minimum positron lifetime of *x* = 1.5% sample was 356.7 ps. The nonlinear variation of *τ*_2_ value with doping concentration could be explained by the competition between the effects of large radius and low valence of Zn ion doping. For VO_2_(B) samples, the substitution of Zn^2+^ with a larger radius would generate lattice distortion and increase vacancy defects, whereas the replacement of Zn^2+^ with a low valence would modify the electron distribution state and decrease the vacancy defects. Under the combined actions of these two factors, when *x* ≤ 1.5%, the valence state effect played a major role resulted in the *τ*_2_ decreasing. When *x* > 1.5%, the radius effect took the lead role, which contributed to the increase of *τ*_2_. The value of *τ*_2_/*τ*_b_ ratio could be used to judge the defect type [26]. The ratio in the ranges of 1.1–1.3, 1.3–1.4, and >1.5 corresponded to a single vacancy, double vacancy and vacancy cluster, respectively. The calculated *τ*_2_/*τ*_b_ values were 1.39, 1.26, 1.13 and 1.11 for the *x* = 0.0%, 0.5%, 1.5% and 3.0% samples, which corresponded to double vacancy, single vacancy, single vacancy and single vacancy, respectively. The variation of *τ*_2_/*τ*_b_ values provided more direct evidence for the double vacancy decomposition caused by Zn doping. As shown in Figure 5, *I*_2_ increased continuously when Zn concentration *x* increased from 0.0 % to 3.0%. However, the rate of increase dropped significantly when *x* > 1.5%. This demonstrated that a moderate amount of Zn doping could increase the defect concentration in the Zn-VO_2_(B) samples, which was due to the structural distortion caused by the substitution of V^4+^ for Zn^2+^ with larger ionic radius. In summary, the defect size and concentration in the Zn-VO_2_(B) samples were greatly affected by Zn doping at the V site.

### 3.5. Electrochemical Performance Analysis

**Figure 6:**
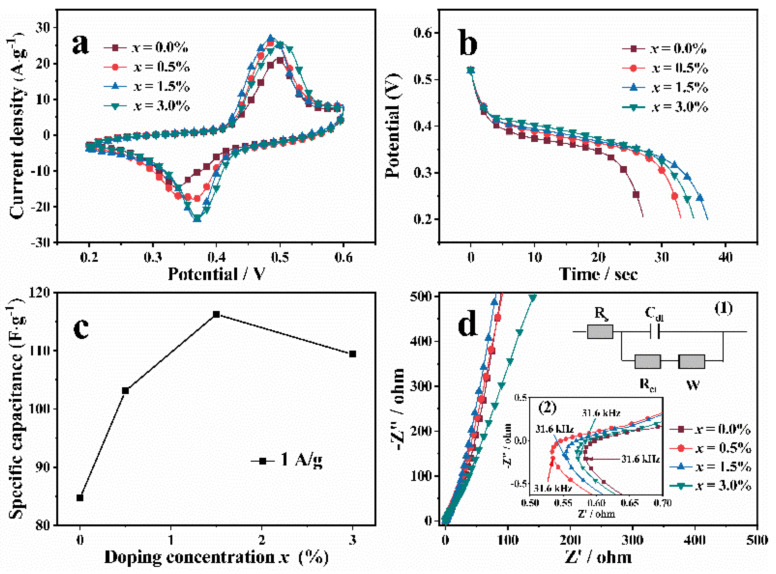
The Electrochemical performance of Zn-VO_2_(B) electrodes with different *x* values: (**a**) CV curves at the scan rate of 100 mV·s^−1^; (**b**) Potential vs. time for discharge profiles at the current density of 1 A·g^−1^; (**c**) Specific capacitance of samples at 1 A·g^−1^; (**d**) Nyquist plots curves; illustration (1) is equivalent circuit diagram; illustration (2) is amplifying high frequency zone.

To investigate the electrochemical performances of the synthesized Zn-VO_2_(B) electrodes, cyclic voltammetry (CV), galvanostatic charge-discharge (GCD) and electrochemical impedance spectroscopy (EIS) were carried out by a three-electrode cell. Figure 6a displayed the CV curves of Zn-VO_2_(B) electrodes with different doping concentration *x* at 100 mV/s from 0.2 V to 0.6 V. The current peaks of oxidation-reduction reactions appeared in the CV curve of all the samples. The symmetry of peak shape indicated the superior reversibility and stability of the Zn-VO_2_(B) electrode, which was conducive to the stable insertion and release of K ion [17]. As shown in Figure 6b, the galvanostatic discharge measurement of the Zn-VO_2_(B) cathode had been taken at 1 A·g^−1^. Compared with the undoped VO_2_(B) cathode, the Zn-VO_2_(B) cathodes had longer discharge time and discharge voltage platform, as depicted in Figure 6b. The above results indicated that Zn doping was conducive to promoting the K^+^ insertion process into vanadium oxide. According to the discharge curves of Zn-VO_2_(B) with different *x*, the sample with *x* = 1.5% had longest discharge time and discharge voltage platform. This phenomenon was consistent with SEM results. The sample with *x* = 0.015 showed the smallest grain size (as shown in Figure 4), which induced the shorter diffusion distance than other samples. The shorter electron transport distance was beneficial to the redox reaction.

The discharge curve can be used to calculate the specific capacitance through the formula [27]

$$C=\frac{2i_{m}\int Vdt}{V^{2}{丨}_{V_{1}}^{V_{2}}}$$

In the above formula, C stands for the specific capacitance, $i_{m}$ is the current density. ∫Vdt is the current integral area, *V* is the potential, *V*_1_ is the initial potential and *V*_2_ is the final potential value. The calculated specific capacitances of Zn-VO_2_(B) with different *x* were displayed in Figure 6c. With increasing Zn content, the specific capacitances increased continuously up to *x* = 1.5%, and decreased rapidly at *x* > 1.5%. The maximum specific capacitance of the *x* = 1.5% sample was 116.56 F·g^−1^. Based on the analysis results of CV, GCD and calculated specific capacitance, it could be found that appropriate amount of Zn doping could effectively improve the electrochemical properties of Zn-VO_2_(B) system. The sample with *x* = 1.5% had the best electrochemical performances compared with other samples. The enhanced mechanism of electrochemical performance could be explained by the defect evolution. Under the combined action of large radius effect and low valence effect, the vacancy defects inside the sample with *x* = 1.5% had the smallest volume and the higher concentration. These small volume vacancy defects were equivalent to many small active sites, which promoted the insertion of alkali metal ions (Li^+^, Na^+^, K^+^) into the electrode material and improvement of the discharge capacity of Zn-VO_2_(B) nanorods.

The EIS was used to study the conductivity and resistance characteristics in the frequency range of 0.01–10^5^ Hz, as shown in Figure 6d. The illustration (1) in Figure 6d showed the simulated equivalent circuit. The W stands for the Warburg impedance; C_dl_ represents the double layer capacitor on the surface of the electrode; R_ct_ is the charge transfer resistance between the electrode and the electrolyte; R_s_ delegates the electrolyte resistance. In the low frequency region, the higher slope of the straight line means smaller ion diffusion resistance [28,29,30]. The *x* = 1.5% sample had a smaller diffusion resistance than other samples. The illustration (2) in Figure 6d showed the amplifying impedance curve in the high-frequency region. The intersection of the impedance curve and the real axis represents the ohmic resistance of the electrolyte and the surface of the electrode, corresponding to R_s_ in the equivalent circuit. Compared with undoped sample, the resistance of Zn-doped VO_2_(B) samples decreased.

**Figure 7:**
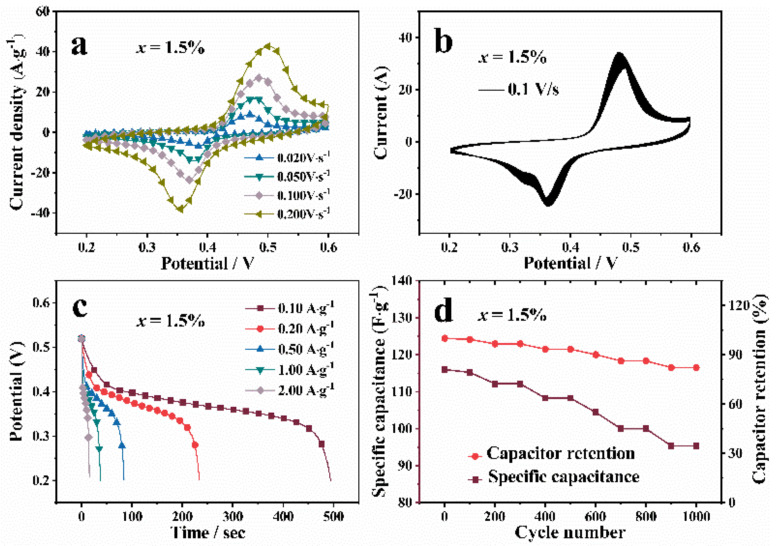
The Electrochemical measurements of Zn-VO_2_(B) sample with *x* = 1.5%: (**a**) CV curves at different scan rates; (**b**) CV curves of 1000 cycles at 100 mV/s scanning rate; (**c**) galvanostatic discharge profiles at different current density; (**d**) cycling performances at 0.1 A·g^−1^.

The *x* = 1.5% sample had the optimum electrochemical performance, and further researchs on *x* = 1.5% sample was carried out, as shown in Figure 7. Figure 7a showed the CV curves of *x* = 1.5% sample at 20 mV/s, 50 mV/s, 100 mV/s and 200 mV/s scanning rate. With the increase of scanning rate, the potentials of the oxidation peak and reduction peak also increased significantly, which was attributed to the increase of overpotential. The peak shape did not change obviously with the increase of scanning rate, which proved that the cathode material had good rate performance. As shown in Figure 7b, the CV curves overlapped well after 1000 cycles at 100 mV/s scanning rate, indicating the good reversibility and cyclic stability of Zn-VO_2_(B) electrodes. Figure 7c showed the galvanostatic discharge curves of Zn-VO_2_(B) with *x* = 1.5% sample at the current density of 0.1 A/g, 0.2 A/g, 0.5 A/g, 1.00 A/g, 2.00 A/g. The discharge time and the specific capacitance of electrodes decreased continuously with the gradual increase of current density. The reduced specific capacitance was attributed to the increase of the ionic resistance coefficient caused by the high current discharge process. The cycle performances could be observed in charge/discharge curves, as shown in Figure 7d. The capacitance retention rate of Zn-VO_2_(B) decreased slightly, but still maintained 88% capacitance retention rate after 1000 cycles, which was superior to pure VO_2_(B) and other cathode materials. Accordingly, Zn doping can efficiently improve the cyclic stability of the Zn-VO_2_(B) electrodes and enable the fast kinetics of K ion. The results showed that the synthesized Zn-VO_2_(B) electrode material had good cycle stability, which was of great significance for the practical application in storage batteries.

## 4. Conclusions

In summary, the Zn-VO_2_(B) samples had been synthesized by the method of solvothermal and further heat treatment. The prepared Zn-VO_2_(B) samples exhibited nanorods structure, offering fast transport paths for K ion together with superior structural stability. The introduction of Zn^2+^ with larger ion radius and low valence could effectively regulate the defect volume and concentration, leading to the improvement of specific capacitance and long cycle performance of nano-VO_2_(B) cathode materials. This work suggested that Zn-doped VO_2_(B) was an efficient and feasible strategy for fabrication of high-performance cathodes and provided experimental support for the potential application of VO_2_(B) in electrode materials of storage batteries.

## Figures and Tables

**Table 1:** The calculated lattice constants (a, b, c, β) and unit cell volumes (V) of the Zn-VO_2_(B) samples.

Doping Concentration	Lattice Parameters				β
x	a(Å)	b(Å)	c(Å)	V(Å^3^)	
0.0%	12.085076	3.692486	6.435457	274.704	106.948
0.5%	12.107583	3.699608	6.451428	276.424	106.952
1.5%	12.103891	3.697008	6.451905	276.200	106.929
3.0%	12.109881	3.699087	6.456372	276.705	106.936

**Table 2:** The positron lifetime parameters *τ*_1_, *τ*_2_, *τ*_3_, *I*_1_, *I*_2_, *I*_3_ and *τ*_b_ of Zn-doped VO_2_(B) with different doping concentration *x*.

Sample	Positron Lifetime/ps			Intensity/%			Bulk Lifetime/ps
*x*	*τ*_1_	*τ*_2_	*τ*_3_	*I*_1_	*I*_2_	*I*_3_	*τ*_b_
0.0%	256.0	433.1	1149.3	58.7545	39.8729	1.3726	310.9707
0.5%	248.9	388.7	1315.7	48.4924	51.1953	0.3123	306.2451
1.5%	222.2	356.7	1277.8	23.2777	75.9608	0.7615	314.7481
3.0%	228.3	361.7	1479.5	20.4139	79.0256	0.5605	324.7799

## Data Availability

The data presented in this study are available on request from the corresponding author.
